# Correction: Impact of clinical severity of stroke on the severity and recovery of visuospatial neglect

**DOI:** 10.1371/journal.pone.0202434

**Published:** 2018-08-10

**Authors:** The PLOS ONE Staff

## Notice of republication

This article was republished on July 30, 2018, to correct for errors in the Data Availability statement introduced during the production process. The publisher apologizes for the errors. Please download this article again to view the correct version. The originally published, uncorrected article and the republished, corrected articles are provided here for reference.

## Supporting information

S1 File Originally published, uncorrected article. (PDF)

S2 File Republished, corrected article. (PDF)
