# Supplementary material for: Correction: Impact of clinical severity of stroke on the severity and recovery of visuospatial neglect
Source: PLoS One. 2018 Aug 10;13(8):e0202434. doi: 10.1371/journal.pone.0202434 (PMC6086433; doi:10.1371/journal.pone.0202434)
Supplement: S1 File — (PDF) [file pone.0202434.s001.pdf]

PLOS ONE

RESEARCH ARTICLE

# Impact of clinical severity of stroke on the severity and recovery of visuospatial neglect

**Tanja C. W. Nijboer**[1,2,3]*, **Caroline Winters**[4,5], **Boudewijn J. Kollen**[6], **Gert Kwakkel**[4,5,7,8]

**1** Utrecht University, Experimental Psychology, Utrecht, the Netherlands, **2** University Medical Center Utrecht, Brain Center Rudolf Magnus, Utrecht, the Netherlands, **3** Center of Excellence for Rehabilitation Medicine, University Medical Center Utrecht and de Hoogstraat Rehabilitation Center, Utrecht, the Netherlands, **4** Department of Rehabilitation Medicine, VU University Medical Center, Amsterdam Movement Sciences, Amsterdam, the Netherlands, **5** Amsterdam Neuroscience Campus, Vrije Universiteit Amsterdam, Amsterdam, the Netherlands, **6** Department of General Practice, University of Groningen, University Medical Center Groningen, Groningen, the Netherlands, **7** Department of Neurorehabilitation, Centre of Rehabilitation and Rheumatology READE, Amsterdam, The Netherlands, **8** Department of Physical Therapy and Human Movement Sciences, Northwestern University, Chicago, Illinois, United States of America

* t.c.w.nijboer@uu.nl

Check for updates

OPEN ACCESS

**Citation:** Nijboer TCW, Winters C, Kollen BJ, Kwakkel G (2018) Impact of clinical severity of stroke on the severity and recovery of visuospatial neglect. PLoS ONE 13(7): e0198755. https://doi.org/10.1371/journal.pone.0198755

**Editor:** Terence J Quinn, University of Glasgow, UNITED KINGDOM

**Received:** November 6, 2017

**Accepted:** May 24, 2018

**Published:** July 2, 2018

**Copyright:** © 2018 Nijboer et al. This is an open access article distributed under the terms of the Creative Commons Attribution License, which permits unrestricted use, distribution, and reproduction in any medium, provided the original author and source are credited.

**Data Availability Statement:** **Note to PAs at accept: query author on involvement of funders and consortium in making data available** The data cannot be made publicly available because patients did not gave explicit their written informed consent in which they agreed to share their data for public use. However, the anonymous data may be requested from Dr. Carel Meskers (C.Meskers@vumc.nl; EXPLICIT trial), or from the author Prof. Dr. Gert Kwakkel (G.Kwakkel@vumc.nl), who will evaluate the request for its purposes in line with the funding organizations (ZonMw), the medical ethical

## Abstract

### Background and purpose

There is growing evidence that visuospatial neglect (VSN) is associated with lower functional performance in other modalities and is not restricted to the lesioned hemisphere alone, and may also affect the non-lesioned hemisphere in severe first-ever strokes. We aimed to investigate the longitudinal association between the severity of VSN, as reflected by the extent of ipsilesional and contralesional spatial attention deficit, and clinical severity of stroke.

### Methods

This is a secondary data analysis with merged data from two prospective cohort studies. Resulting in 90 patients and 8 longitudinal measurements at 1, 2, 3, 4, 5, 8, 12, and 26 weeks post-stroke onset. A letter cancellation test (LCT) was used as the primary outcome measure to demonstrate presence and severity of VSN. The clinical severity of stroke was classified using the Bamford Classification.

### Results

No significant association between clinical severity and the number of ipsilesional, as well as contralesional, omissions on the LCT was observed. Recovery of VSN at the contralesional hemiplegic, as well as ipsilesional non-hemiplegic side, was only dependent on 'time' as a reflection of spontaneous neurobiological recovery post-stroke. The recovery of the ipsilesional extension of VSN was significantly slower for the total anterior circulation infarct (TACI) group compared to the non-TACI group.

committee of Leiden University Medical Center and the collaborating consortium of the EXPLICIT-stroke trial.

**Funding:** Supported by a grant from NWO (Netherlands Organization for Scientific Research; grant 451-10-013 to TCWN, and by the EXPLICIT-stroke grant of ZonMw (grant 89000001) and 4D-EEG (ERC advanced grant 291339-4D-EEG) to GK. The funders had no role in study design, data collection and analysis, decision to publish, or preparation of the manuscript.

**Competing interests:** The authors have declared that no competing interests exist.

## Conclusions

Larger strokes have a significant negative impact on recovery of visual attention at the non-hemiplegic side. No clinical determinants that regulate spontaneous time-dependent recovery of VSN were found. While early 'stroke severity' has been regarded as a strong predictor of functional outcome at a group level, other prognostic factors (demographic, stroke related) need to be determined.

## Clinical trial registration

EXPLICIT-stroke Trial: http://www.trialregister.nl/trialreg/admin/rctview.asp?TC=1424

Stroke Intensity Trial: http://www.trialregister.nl/trialreg/admin/rctview.asp?TC=1665

## Introduction

Visuospatial neglect (VSN) is a frequent disorder following stroke, leaving patients with impaired or even lost awareness for contralesional stimuli and/or events (i.e. side of space opposite to the lesioned hemisphere). In very severe cases of VSN, the deficit may also encompass stimuli and/or events at the ipsilesional side, in other words, the same side of space as the lesioned hemisphere. Either due to hypo-attention to the contralesional field [1, 2], hyper-attention to the ipsilesional field [3] or attentional imbalance and hemispheric rivalry [4, 5]. At the behavioural level this results in comparable observations, leaving patients with VSN with a limited magnitude of space that they are aware off.

Although spontaneous neurobiological recovery of VSN occurs naturally in most patients within the first 10–12 weeks post-stroke onset [6], it remains present in up to 40% in patients with severe stroke [6, 7]. Several cohort studies with repeated measures in time suggested that VSN is negatively associated with magnitude of recovery of other neurological impairments such as motor recovery post-stroke [8, 9], and functional outcomes [6, 10]. In addition, there is growing evidence that VSN is associated with lower functional performance in other neurological modalities [9] and not restricted to the lesioned hemisphere alone. VSN, however, may also affect the so called non-lesioned hemisphere in (very) severe first-ever strokes [11], most likely due to interhemispheric white matter disconnection between both hemispheres. For accurate prognosis, more insight is necessary in the complex interaction between severity of stroke and attention deficits of both the contralesional and the ipsilesional side early post-stroke onset.

Therefore, the primary aim of the current study was to investigate the association between the severity of VSN after right hemisphere stroke, as reflected by the extent of ipsilesional visuospatial attention deficit (besides the contralesional spatial attention deficit) and clinical severity of stroke indicated with the Bamford Classification [12]. The clinical severity of stroke is strongly predictive of functional outcome on a group level [13] and was categorised as total anterior circulation infarct (TACI) versus non-TACI (see 2.3 Outcome measures). The second aim was to investigate the longitudinal association between the time course of recovery of the ipsilesional visuospatial deficit, recovery of contralesional visuospatial attention deficit, and clinical severity of stroke (TACI versus non-TACI). We hypothesized that a contralesional visuospatial attention deficit is comparable between large and moderate strokes (TACI versus non-TACI), that especially the magnitude of the ipsilesional visuospatial deficit is associated

with stroke severity and that the time course of spontaneous neurobiological recovery in terms of VSN is prolonged in case of larger strokes (TACI).

# Methods

## Patients

This is a secondary data analysis with data merged from two prospective cohort studies: the EXPLICIT-stroke trial [14, 15](NTR, www.trialregister.nl, TC1424) and the Stroke Intensity trial [16, 17].

Only patients with VSN were included for the statistical analyses in the present study. From the 260 stroke patients from both cohort studies, 90 patients with a first-ever, ischemic, right-hemisphere stroke *and* VSN (as measured with a letter cancellation test, see 2.3 Outcome measures) were included in the present study (see also [18]). Informed consent was obtained in accordance with the declaration of Helsinki (2013). The EXPLICIT-stroke Trial was registered in the Dutch Trial Registry (NTR, www.trialregister.nl, TC1424) and approved by the by the Medical Ethics Review Committees of Leiden University Medical Center (No. P08.035) and the Dutch Central Committee on Research Involving Human Subjects (CCMO: No. NL21396.058.08). The Intensity Trial was registered in the Dutch Trial Registrey (NTR, www.trialregister.nl, TC1665) and approved by the local Ethical Committee of the VU University Medical Centre, Amsterdam, the Netherlands (https://www.vumc.nl/afdelingen/METc/METc/). The authors confirm that all related trials for this intervention were registered.

## Procedure

For both trials, the research protocols were implemented within 14 days post-stroke onset. The interventions were focussed at functional motor recovery. Final outcome was defined at 26 weeks for the EXPLICIT-stroke trial and 52 weeks for the Stroke Intensity trial. For the current study, this resulted in 8 longitudinal, weekly measurements at weeks 1 up to 5, and follow-up measurements at weeks 8, 12, and 26. All outcome measures were obtained during these measurements.

## Outcome measures

In the present study, the letter cancellation test (LCT) was used as the primary outcome measure to demonstrate presence and severity of VSN. Here, patients had to cancel O-s among other letters on a sheet of A4 paper containing 20 O-s on the left side, 20 O-s on the right side, among 425 distractors in total [19]. Both targets and distractors were randomly arranged throughout the page. The difference between the number of omissions on the left versus right side of the paper was used to indicate VSN. To clarify, [i.e. an asymmetry score of at least 2 contra versus ipsilesional omissions, 6].

The clinical severity of stroke was classified using the Bamford classification [12]. This classification distinguishes reliably and validly between a TACI, partial anterior circulation infarct (PACI), lacunar anterior circulation infarct (LACI) or posterior circulation infarct (POCI) [12]. With respect to the aims of this study, we focus on TACI and non-TACI (i.e., LACI or PACI). Diagnosis of TACI (affecting the entire anterior circulation supplying one hemisphere) requires patients to show (1) hemiparesis of the face, arm and/or leg, (2) homonymous hemianopia, and (3) cognitive deficits, such as VSN [12]. All three symptoms are needed for the classification of TACI. In contrast, in patients with smaller strokes affecting only part of the anterior circulation supplying one hemisphere, only two of the abovementioned symptoms are needed for the classification (non-TACI). In the present study patients in the non-TACI group

had hemiparesis of the face, arm and/or leg and VSN. The Bamford classification is found to be a reliable [20] and valid classification associated with findings from CT or MRI-scans [12, 21], showing predictive validity with respect to meaningful outcomes such as ADL [12, 22].

The patients' medical records were also reviewed to capture the following relevant data: age, sex, and time post-stroke onset. Additionally, intervention type (arm training, leg training, immobilisation, EXPLICIT-stroke treatment, EXPLICIT-stroke control) and clinical assessments were noted: cognitive impairments as measured with the mini mental state examination (MMSE), synergistic motor control of the paretic arm as measured with the Fugl-Meyer assessment (FMA-arm), motor strength of the paretic arm as measured with the motricity index (MI-arm), and independence in activities of daily living as measured with the Barthel index (BI).

The MMSE [23] examines orientation, memory, attention, calculation, language, and construction functions. Scores vary from 0 (severe cognitive impairments) up to 30 (no cognitive impairments). In general, a score of less than 24 is considered as cognitive impairment.

The FMA-arm [24] is a stroke-specific, performance based impairment index, designed to assess motor functioning in patients with post-stroke hemiplegia. It contains 33 items scored on a 3-point scale (i.e. 0, 1, and 2 points; range 0–66 points; 66 reflects normal motor function).

The MI-arm [25] consists of three items for the arms (i.e. pinch grip, elbow flexion, shoulder abduction). Scores range from 0–100, with 100 points reflecting normal motor function (ordinal 6-point scale (i.e. 0, 11, 19, 22, 26, and 33 points) per item +1).

The BI [26] measures the extent of independence and mobility in ADL, i.e. feeding, bathing, grooming, dressing, bowel and bladder control, toileting, chair transfer, ambulation, and stair climbing. Scores range from 0 (completely dependent) up to 20 (completely independent).

### Statistical analyses

First, demographic and clinical stroke characteristics were compared between the two groups, patients with a TACI versus patients with a non-TACI, using non-parametric tests.

Next, the regression coefficient was estimated for the association between the predictor clinical severity (i.e. TACI versus non-TACI) and outcome (i.e. contralesional or ipsilesional omissions) adjusted for study population. In a separate model this association was also adjusted for time. Moreover, in this latter model interaction terms between clinical severity and time were added to investigate whether this severity was dependent on time. The data structure was clearly hierarchical, as repeated observations (level 1) were nested within patients (level 2). The data analysis required implementation of multilevel random coefficient analysis, which was performed using MLWIN version 2.26. The restricted iterative generalised least-squares (RIGLS) estimation procedure was used to estimate the regression coefficients of the derived model. Assumptions required for conducting regression analyses were assessed by inspecting normal probability plots and plots of standardized residuals versus predicted values. We controlled for clinical trial (because studies had different inclusion criteria) in the multilevel analyses. The time-dependency of both ipsilesional and contralesional VSN data was investigated by using random coefficient analyses, corrected for type of intervention. For all tests, a two-tailed significance level of .05 was used. The Wald-test was used to obtain p-values for the regression coefficients.

## Results

### Demographic and stroke characteristics

An overview of demographic and stroke characteristics at baseline is given in Table 1. Both groups (TACI versus non-TACI) were comparable with respect to age ($U = 861.5$, $z = -1.034$, $p = .301$), sex ($\chi^2 = 1.600$, $p = .206$), time post-stroke onset ($U = 978$, $z = -.082$, $p = .934$),

**Table 1. Demographical and stroke characteristics of patients with VSN, per group at baseline (TACI versus non-TACI).**

| Clinical variables | Results TACI (SD) | Results Non-TACI (SD) |
|---|---|---|
| Group size | 38 | 52 |
| Age in years | 62.42 (11.92) | 60.06 (11.92) |
| Sex (male) | 52.6% | 59.6% |
| Time post-stroke in days | 7.29 (2.56) | 7.35 (2.62) |
| Study | | |
| *Stroke Intensity trial* [16, 27] | 81.3% | 11.5% |
| *EXPLICIT-stroke* trial[14,15] | 18.8% | 88.5% |
| MMSE (0–30) | 25.81 (2.42) | 26.62 (2.60) |
| BI (0–20) | 5.00 (4.09) | 6.02 (4.29) |
| FMA-arm (0–66) | 9.71 (11.33) | 11.27 (20.05) |
| MI-arm (0–100) | 10.78 (21.53) | 15.04 (26.00) |
| LCT contralesional omissions (0–20) | 16.36 (4.59) | 13.43 (6.81) |
| LCT ipsilesional omissions (0–20) | 6.81 (6.40) | 6.00 (6.45) |

BI: Barthel Index; FMA-arm: Fugl-Meyer Assessment arm; LCT: Letter Cancellation Test; MI: Motricity Index; TACI: total anterior circulation infarct according to the Bamford classification; non-TACI: other classification than TACI, according to the Bamford classification [12].

https://doi.org/10.1371/journal.pone.0198755.t001

MMSE (U = 681.5, z = -1.709, p = .087), BI (U = 603.5, z = -1.202, p = .229), FMA-arm (U = 646, z = -1.132, p = .258), MI-arm (U = 702.5, z = -1.075, p = .282), and contralesional (U = 530, z = -1.450, p = .147) and ipsilesional omissions (U = 526, z = -.980, p = .327). Type of treatment was unequally distributed across groups (overall: $\chi^2$ = 15.00, p = .005; TACI: $\chi^2$ = 11.737, p = .019; non-TACI: $\chi^2$ = 52.231, p < .001).

In Table 2, the median number of omissions (and IQR) on the letter cancellation test is given per side per week, split for group (TACI versus non-TACI). In Fig 1, the distributions of the number of omissions are given per side per group.

## Random coefficient analysis

**Ipsilesional VSN.** Overall, no significant association was observed between clinical severity (TACI versus non-TACI) and number of ipsilesional omissions measured with the LCT (Table 3). However, the association between clinical severity and number of ipsilesional omissions appeared dependent upon the time of measurement post-stroke. Corrected for type of intervention, the number of ipsilesional omissions decreased weekly with 0.14 during the first 26 weeks, whereas its relation with clinical severity decreased with 0.06 (Table 4). In other words, fewer ipsilesional omissions were observed with increasing time since the stroke event.

**Contralesional VSN.** Overall, no significant association was observed between clinical severity (TACI versus non-TACI) and number of contralesional omissions (Table 3). Also, no time-dependent association between clinical severity and number of contralesional omissions was observed (Table 4). In this model, only a significant association was observed between number of contralesional omissions and time post-stroke onset; with each weekly and follow-up measurement, the number of contralesional omissions dropped with 0.31 (Table 3).

## Discussion

The current study shows no significant overall association between clinical severity assessed within the first 2 weeks post-stroke onset and the number of ipsilesional, as well as contralesional

Table 2. The median number of omissions (and IQR), per side (ipsilesional versus contralesional), per week, split for group (TACI versus non-TACI).

| Time post-stroke onset | Number of omissions on the LCT for TACI (IQR) (N = 38) | Number of omissions on the LCT for Non-TACI (IQR) (N = 52) |
|---|---|---|
| Ipsilesional | | |
| 1 week | 3.5 (1.75–14.25) | 3 (0–11) |
| 2 weeks | 2 (0–10.5) | 1 (0–5) |
| 3 weeks | 1 (0–7) | 1 (0–5) |
| 4 weeks | 1 (0–6) | 0 (0–3) |
| 5 weeks | 1 (0–2) | 0 (0–2) |
| 8 weeks | 0 (0–2) | 0 (0–1) |
| 12 weeks | 0 (0–1) | 0 (0–1) |
| 26 weeks | 0 (0) | 0 (0–1) |
| Contralesional | | |
| 1 week | 18 (14–20) | 16 (7–20) |
| 2 weeks | 11 (7–20) | 9 (2–19) |
| 3 weeks | 12 (4–20) | 6 (2–15) |
| 4 weeks | 8 (3–20) | 6 (2–14) |
| 5 weeks | 8 (3–16) | 3.5 (1–8) |
| 8 weeks | 4 (1–10) | 3 (.5–7.5) |
| 12 weeks | 3 (0.25–6) | 3 (1–7.25) |
| 26 weeks | 2 (0–7.5) | 2 (0–4) |

Contralesional: side of space opposite to the lesioned hemisphere); ipsilesional: side of space on the same side as the lesioned hemisphere; LCT: letter cancellation test.

https://doi.org/10.1371/journal.pone.0198755.t002

omissions measured, with the LCT. Recovery of VSN at the contralesional hemiplegic, as well as ipsilesional non-hemiplegic side, was only dependent on 'time' as a reflection of spontaneous neurobiological recovery post-stroke. Recovery of VSN at the contralesional and ipsilesional side was comparable between both groups, yet a trend toward lower omission scores was observed for the ipsilesional side only.

Irrespective of initial severity of stroke, only progress of time, as a reflection of spontaneous neurobiological recovery [28], seems to be the only factor responsible for recovery of VSN post-stroke. To date, this time course of neurological recovery is observed for several neurological impairments such as speech [29], upper [30] and lower limb motor impairment [31] as well VSN [18]. In the recent study by Winters et al [18] on the same cohort, 80 out of the 90 right hemispheric strokes followed a 80% proportional recovery rule for VSN after stroke [18]. The 10 non-fitters who failed to show any spontaneous neurological recovery for VSN also failed to show spontaneous recovery for other modalities such as motor impairment [18]. This finding suggests that the time course of spontaneous neurobiological recovery including that for VSN is driven by a common poorly understood mechanism which is already defined within the first weeks post-stroke onset [32]. Similarly, Winters et al also showed that the Bamford classification score was not a significant determinant for both patients who did or did not fit the fixed proportional recovery rule [18]. Aforementioned findings support the theory of Von Monakov with respect to diaschisis, the temporary loss of excitability of neurons remote from the original lesion [33–35]. The core of the theory is that the loss of remote excitability may explain clinical symptoms that cannot be directly related to the lesion, that it resolves over time and that the severity of behavioural consequences is strongly correlated with the severity of diaschisis [33–35]. Connectional diaschisis encompasses changes in connectivity between

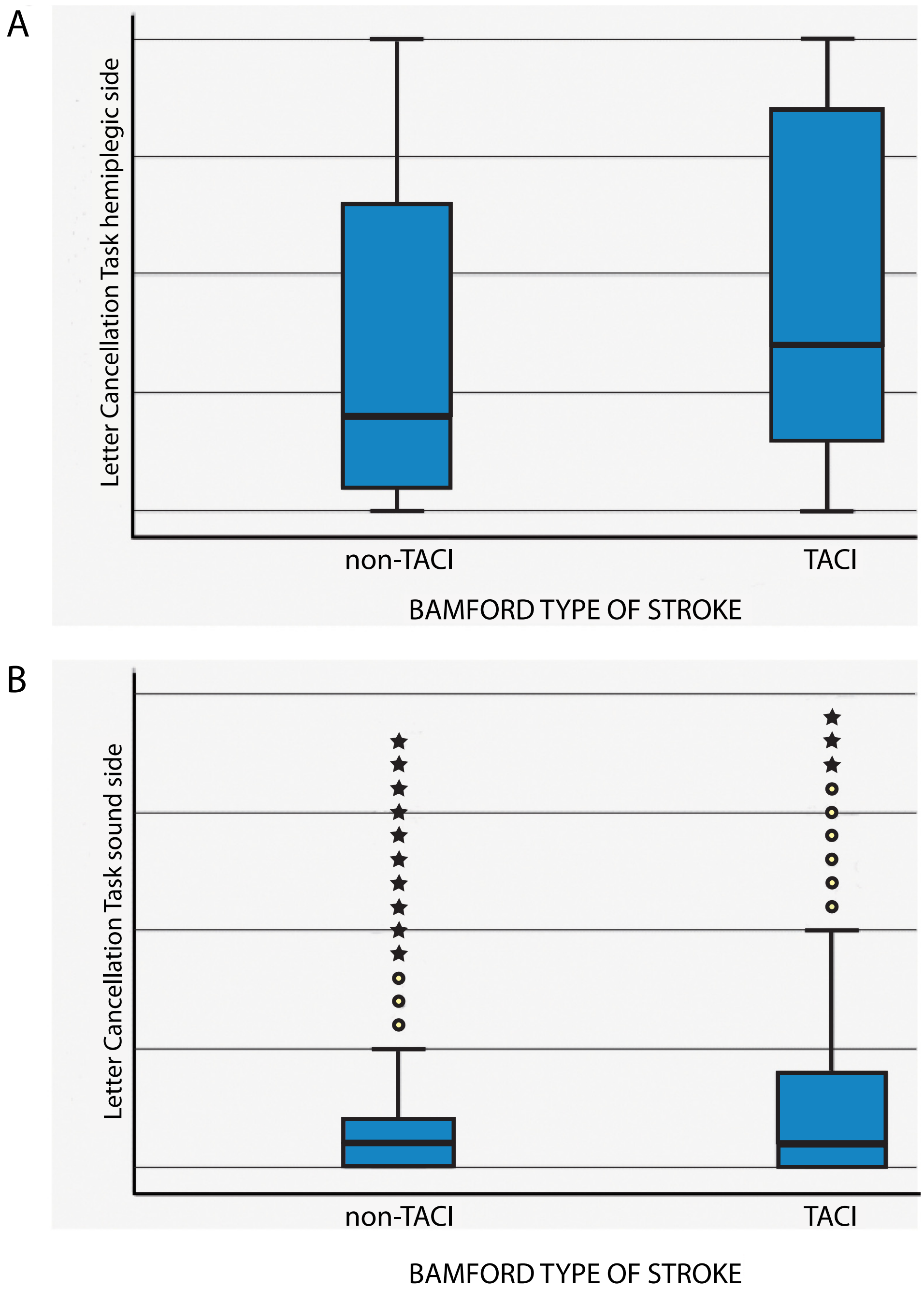


**Fig 1. Distributional characteristics of the scores on the letter cancellation task per group (TACI versus non-TACI), split for side: a. contralesional hemiplegic side, b. ipsilesional 'sound' side.**

https://doi.org/10.1371/journal.pone.0198755.g001

affected areas (including the lesioned area) and even the ipsilesional hemisphere [36]. Unfortunately, we did not find clinical determinants that predicted the time course of spontaneous neurobiological recovery [37, 38]. Acknowledging that this time course of spontaneous recovery of neglect is proportionally fixed for most patients [18], 2017), the factors that identify those stroke victims that fail to follow this proportional recovery rule is regarded as one the main targets for future recovery studies [37]. While early 'stroke severity' has been regarded as

**Table 3. Multilevel unstandardized regression coefficients, confidence intervals (CI) and level of significance for the association between clinical severity and time-dependent recovery of ipsilesional and contralesional omissions on the letter cancellation test during the first 26 weeks post-stroke, corrected for trial (N = 90).**

| Outcome measure | β value | 95% CI | P-value |
|---|---|---|---|
| Ipsilesional omissions | | | |
| Clinical severity | 0.89 | -1.04–2.83 | .37 |
| Contralesional omissions | | | |
| Clinical severity | 1.92 | -1.24–5.07 | .23 |

https://doi.org/10.1371/journal.pone.0198755.t003

a strong predictor of functional outcome at a group level, other prognostic factors (e.g., demographic, genetic phenotype as well as stroke related) need to be determined [13]. This stresses the urgency of finding other biomarkers by means of animal [13, 32, 38, 39], molecular, and/or neuro-imaging studies [13, 32, 38].

Even though the present study reflects one of the largest patient cohorts suffering from VSN post-stroke, some limitations need to be addressed. First, we did not have direct measures for lesion size and lesion location. Even though the Bamford classification is a widely used, reliable and valid clinical measure [12], it obviously lacks the level of detail of the volume of the lesion that neuro-imaging measures can display. In the same line of reasoning, not only cortical but also subcortical involvement (integrity of white matter) is a likely candidate for significant biomarkers, related to severity of stroke [32, 38]. Second, only right-hemisphere, first-ever stroke patients were included. A recent study by Ten Brink et al [40] indicated that although the core component of VSN—the lateralised attention deficit—was more severe in left-sided compared to right-sided VSN, there was large overlap in clinical outcomes and impact of VSN on for example motor impairment and independence during ADLs. Given the subset of VSN patients in the present study, it remains unknown whether current results are hemisphere related (i.e. restricted to the right hemisphere or not). Third, VSN was indicated with only one pen-and-paper test. As VSN is a very heterogeneous syndrome, it is likely that a subset of patients who were excluded from the original studies, based on their performance on the LCT, still have VSN. For future studies, it would be important to use different tests for VSN, also to verify whether the clinical manifestations (and range of severity herein) are comparable with respect to, for example, severity of stroke, spontaneous time-dependent recovery, etc. Somewhat related to this limitation, only asymmetry scores were used as the outcome measure and not, for example, a continuous measure of severity such as the center of cancellation. The center of cancellation not only takes into account the number of omissions, but also their specific location, resulting in one outcome measure indicating severity. Fourth, no other

**Table 4. Multilevel unstandardized regression coefficients, confidence intervals (CI) and level of significance for the association between clinical severity and time-dependent recovery of ipsilesional and contralesional omissions on the letter cancellation test during the first 26 weeks post-stroke, corrected for trial (N = 90).**

| Outcome measure | β value | 95% CI | P-value |
|---|---|---|---|
| Ipsilesional omissions | | | |
| Clinical severity | 1.63 | -0.39–3.66 | .11 |
| Time | -0.14 | -0.18–-0.10 | < .001 |
| Clinical severity*time | -0.06 | -0.12–-0.002 | .04 |
| Contralesional omissions | | | |
| Clinical severity | 3.12 | -0.15–6.38 | .06 |
| Time | -0.31 | -0.37–-0.25 | < .001 |
| Clinical severity*time | -0.07 | -0.15–0.02 | .15 |

https://doi.org/10.1371/journal.pone.0198755.t004

data on cognitive impairment was available besides the MMSE as a cognitive screener. Even though both groups showed comparable scores on the MMSE—and above the cut-off value as an indication for cognitive impairment -, we can not rule out that patients would also have other cognitive impairment. Fifth, data from two trials with repeated measurements in time were merged. Even though the inclusion criteria for both trials were largely comparable and we used additional inclusion criteria for this study, it turned out that the more severe stroke were mainly included from the Stroke Intensity trial [16], where the non-TACI patients were largely included from the EXPLICIT-stroke trial [15]. Although we included trial type as a covariate in the analyses, the asymmetrical distribution of participants from the original studies in the newly created cohort might have influenced the outcome. Last, no data was collected in the acute phase—within the first 72 hours post-stroke onset—as initial assessments were performed approximately 7 days post (SD 2.6 days) stroke onset on average. It might be that the largest, most significant interaction effects between, on the one hand, severity of stroke, and, on the other hand, severity of VSN are found in the acute phase post stroke.

## Acknowledgments

We like to thank Rinkse Nijland for her support with collecting and registering the measurements for the EXPLICIT-stroke trial.

## Author Contributions

**Conceptualization:** Tanja C. W. Nijboer, Caroline Winters, Boudewijn J. Kollen, Gert Kwakkel.

**Data curation:** Gert Kwakkel.

**Formal analysis:** Tanja C. W. Nijboer, Boudewijn J. Kollen.

**Funding acquisition:** Gert Kwakkel.

**Investigation:** Gert Kwakkel.

**Methodology:** Tanja C. W. Nijboer, Caroline Winters, Boudewijn J. Kollen, Gert Kwakkel.

**Project administration:** Gert Kwakkel.

**Resources:** Gert Kwakkel.

**Visualization:** Tanja C. W. Nijboer, Boudewijn J. Kollen.

**Writing – original draft:** Tanja C. W. Nijboer.

**Writing – review & editing:** Tanja C. W. Nijboer, Caroline Winters, Boudewijn J. Kollen, Gert Kwakkel.

## References

1. Halligan PW, Marshall JC. The history and clinical presentation of neglect. In: Robertson IH, Marshall JC, editors. Unilateral neglect; clinical and experimental studies. Hove, UK: Lawrence Erlbaum Associates; 1993.
2. Heilman KM, Van Den Abell T. Right hemispheric dominance for attention: the mechanism underlying hemispheric asymmetries of inattention (neglect). Neurology. 1980; 30:327–30. PMID: 7189037
3. Koch G, Oliveri M, Cheeran B, Ruge D, Lo Gerfo E, Salerno S, et al. Hyperexcitability of parietal-motor functional connections in the intact left-hemisphere of patients with neglect. Brain. 2008; 131(12):3147–55.
4. Kinsbourne M. Orientational bias model of unilateral neglect: Evidence from attentional gradients within hemispace. In: Robertson IH, Marshall JC, editors. Unilateral neglect: Clinical and experimental studies. Hove, UK: Erlbaum; 1993. p. 63–86.

5. Kinsbourne M. Mechanisms of unilateral neglect. In: Jeannerod M, editor. Neurophysiological and neuropsychological aspects of spatial neglect. Amsterdam: Elsevier Science Publishers; 1987. p. 69–86.
6. Nijboer TCW, Kollen BJ, Kwakkel G. Time course of visuospatial neglect early after stroke: a longitudinal cohort study. Cortex. 2013; 49(8):2021–7. https://doi.org/10.1016/j.cortex.2012.11.006 PMID: 23332473
7. Kalra L, Perez I, Gupta S, Wittink M. The influence of visual neglect on stroke rehabilitation. Stroke. 1997; 28(7):1386–91. PMID: 9227688
8. Nijboer TCW, Kollen BJ, Kwakkel G. The impact of recovery of visuo-spatial neglect on motor recovery of the upper paretic limb after stroke. PLoS One. 2014; 9(6):e100584. https://doi.org/10.1371/journal.pone.0100584 PMID: 24950224
9. Corbetta M, Kincade MJ, Lewis C, Snyder AZ, Sapir A. Neural basis and recovery of spatial attention deficits in spatial neglect. Nat Neurosci. 2005; 8(11):1603–10. https://doi.org/10.1038/nn1574 PMID: 16234807
10. Katz N, Hartman-Maeir A, Ring H, Soroker N. Functional disability and rehabilitation outcome in right hemisphere damaged patients with and without unilateral spatial neglect. Arch Phys Med Rehab. 1999; 80(4):379–84.
11. Lunven M, Thiebaut de Schotten M, Bourlon C, Duret C, Migliaccio R, Rode G, et al. White matter lesional predictors of chronic visual neglect: a longitudinal study. Brain. 2015; 138:746–60. https://doi.org/10.1093/brain/awu389 PMID: 25609686
12. Bamford J, Sandercock P, Dennis M, Burn J, Warlow C. Classification and natural history of clinically identifiable subtypes of cerebral infarction. Lancet. 1991; 337(8756):1521–6. PMID: 1675378
13. Kwakkel G, Lannin NA, Borschmann K, English C, Ali M, Churilov L, et al. Standardized measurement of sensorimotor recovery in stroke trials: Consensus-based core recommendations from the Stroke Recovery and Rehabilitation Roundtable. International Journal of Stroke. 2017; 12(5):451–61. https://doi.org/10.1177/1747493017711813 PMID: 28697709
14. Kwakkel G, Meskers CG, van Wegen EE, Lankhorst GJ, Geurts AC, van Kuijk AA, et al. Impact of early applied upper limb stimulation: the EXPLICIT-stroke programme design. BMC Neurol. 2008; 8(49).
15. Kwakkel G, Winters C, van Wegen EE, Nijland RH, van Kuijk AA, Visser-Meily A, et al. Effects of Unilateral Upper Limb Training in Two Distinct Prognostic Groups Early After Stroke: The EXPLICIT-Stroke Randomized Clinical Trial. Neurorehabil Neural Repair. 2016; 30(9):804–16. https://doi.org/10.1177/1545968315624784 PMID: 26747128
16. Kwakkel G, Wagenaar RC, Twisk JW, Lankhorst GJ, Koetsier JC. Intensity of leg and arm training after primary middle-cerebral-artery stroke: a randomised trial. Lancet. 1999; 354:191–6. https://doi.org/10.1016/S0140-6736(98)09477-X PMID: 10421300
17. Kwakkel G, Kollen BJ, Wagenaar RC. Long term effects of intensity of upper and lower limb training after stroke: a randomised trial. Journal of Neurology, Neurosurgery & Psychiatry. 2002; 72(4):473–9.
18. Winters C, van Wegen EE, Daffertshofer A, Kwakkel G. Generalizability of the maximum proportional recovery rule to visuospatial neglect early poststroke. Neurorehabil Neural Repair. 2017; 31(4):334–42. https://doi.org/10.1177/1545968316680492 PMID: 27913798
19. Rasquin S, Ooms N, van de Sande P, Beers K, Schmand B. Validiteit en referentie gegevens van een visueel—ruimtelijke neglecttest: de o—zoektest. Tijdschrift voor neuropsychologie. 2009; 3:44–54.
20. Smith CJ, Emsley HC, Libetta CM, Hughes DG, Drennan RF, Vail A, et al. The Oxfordshire Community Stroke Project classification in the early hours of ischemic stroke and relation to infarct site and size on cranial computed tomography. Journal of Stroke and Cerebrovascular Diseases. 2001; 10(5):205–9. https://doi.org/10.1053/jscd.2001.29825 PMID: 17903825
21. Bamford J, Sandercock P, Jones L, Warlow C. The natural history of lacunar infarction: the Oxfordshire Community Stroke Project. Stroke. 1987; 18(3):545–51. PMID: 3590244
22. Bamford J, Sandercock PA, Warlow CP, Slattery J. Interobserver agreement for the assessment of handicap in stroke patients. Stroke. 1989; 20(6):828. PMID: 2728057
23. Folstein MF, Folstein SE, McHugh PR. "Mini-mental state". A practical method for grading the cognitive state of patients for the clinician. J Psychiat Res. 1975; 12:189–98. PMID: 1202204
24. Fugl-Meyer AR, Jääskö L, Leyman I, Olsson S, Steglind S. The post-stroke hemiplegic patient. 1. a method for evaluation of physical performance. Scandinavian Journal of Rehabilitation Medicine. 1975; 7(1):13–31. PMID: 1135616
25. Collin C, Wade D. Assessing motor impairment after stroke: a pilot reliability study. Journal of Neurology, Neurosurgery & Psychiatry. 1990; 53(7):576–9.
26. Collin C, Wade DT, Davies S, Horne V. The Barthel ADL Index: a reliability study. Int Disabil Stud. 1988; 10(2):61–3. PMID: 3403500

27. Kwakkel G, Kollen BJ, van der Grond J, Prevo AJ. Probability of regaining dexterity in the flaccid upper limb: impact of severity of paresis and time since onset in acute stroke. Stroke. 2003; 34(9):2181–6. https://doi.org/10.1161/01.STR.0000087172.16305.CD PMID: 12907818

28. Kwakkel G, Kollen B, Twisk J. Impact of time on improvement of outcome after stroke. Stroke. 2006; 37:2348–53. https://doi.org/10.1161/01.STR.0000238594.91938.1e PMID: 16931787

29. Lazar RM, Minzer B, Antoniello D, Festa JR, Krakauer JW, Marshall RS. Improvement in aphasia scores after stroke is well predicted by initial severity. Stroke. 2010; 41(7):1485–8. https://doi.org/10.1161/STROKEAHA.109.577338 PMID: 20538700

30. Winters C, van Wegen EE, Daffertshofer A, Kwakkel G. Generalizability of the proportional recovery model for the upper extremity after an ischemic stroke. Neurorehabil Neural Repair. 2015; 29(7):614–22. https://doi.org/10.1177/1545968314562115 PMID: 25505223

31. Veerbeek JM, Langbroek-Amersfoort AC, van Wegen EE, Meskers CG, Kwakkel G. Effects of Robot-Assisted Therapy for the Upper Limb After Stroke. Neurorehabil Neural Repair. 2017; 31(2):107–21. https://doi.org/10.1177/1545968316666957 PMID: 27597165

32. Ward NS. Restoring brain function after stroke—bridging the gap between animals and humans. Nature Reviews. 2017; 13:244–55.

33. Andrews RJ. Transhemispheric diaschisis. A review and comment. Stroke. 1991; 22(7):943–9. PMID: 1853416

34. Feeney DM, Baron JC. Diaschisis. Stroke. 1986; 17(5):817–30. PMID: 3532434

35. Von Monakow C. Diaschisis. Baltimore, MD: Penguin; 1969.

36. Carrera E, Tononi G. Diaschisis: past, present, future. Brain. 2014; 137:2408–22. https://doi.org/10.1093/brain/awu101 PMID: 24871646

37. Bernhardt J, Hayward KS, Kwakkel G, Ward NS, Wolf SL, Borschmann K, et al. Agreed Definitions and a Shared Vision for New Standards in Stroke Recovery Research: The Stroke Recovery and Rehabilitation Roundtable Taskforce. Neurorehabil Neural Repair. 2017; 31(9):793–9. https://doi.org/10.1177/1545968317732668 PMID: 28934920

38. Boyd LA, Hayward KS, Ward NSS, C. M., Rosso C, Fisher RJ, Carter AR, et al. Biomarkers of stroke recovery: Consensus-based core recommendations from the Stroke Recovery and Rehabilitation Roundtable International Journal of Stroke. 2017; 12(5):480–93. https://doi.org/10.1177/1747493017714176 PMID: 28697711

39. Corbett D, Carmichael ST, Murphy TH, Jones TA, Schwab ME, Jolkkonen J, et al. Enhancing the alignment of the preclinical and clinical stroke recovery research pipeline: Consensus-based core recommendations from the Stroke Recovery and Rehabilitation Roundtable translational working group. International Journal of Stroke. 2017; 12(5):462–71. https://doi.org/10.1177/1747493017711814 PMID: 28697710

40. Ten Brink AF, Biesbroek JM, Kuijf HJ, Van der Stigchel S, Oort Q, Visser-Meily JMA, et al. The right hemisphere is dominant in organization of visual search—a study in stroke patients. Behavioural Brain Research. 2016; 304:71–9. https://doi.org/10.1016/j.bbr.2016.02.004 PMID: 26876010
